# Maternal-fetal bonding during the COVID-19 pandemic

**DOI:** 10.1186/s12884-021-04272-9

**Published:** 2021-12-28

**Authors:** Amanda Koire, Leena Mittal, Carmina Erdei, Cindy H. Liu

**Affiliations:** 1 Department of Psychiatry, Brigham and Women’s Hospital, Boston, MA USA; 2 Harvard Medical School, Boston, MA USA; 3 Department of Newborn Medicine, Brigham and Women’s Hospital, Boston, MA USA

**Keywords:** Mental health, Pregnancy, Depression, Anxiety, Stress, Resilience, Attachment, Life events, Parenting, Distress

## Abstract

**Abstract:**

**Background:**

The pregnant population experienced unique COVID-19 physical and psychosocial stressors such as direct health concerns related to the virus and loss of access to resources since the COVID-19 emerged as a global pandemic in early 2020. Despite these COVID-19-related stress and concerns, the maternal experience of bonding with their unborn children has not been well studied. This work aimed to study the association between mental health history, current mental health symptoms, psychological factors, COVID-19-related worries, and self-reported maternal-fetal bonding of pregnant women.

**Methods:**

This online, survey-based cross-sectional study focused on women pregnant during the pandemic and assessed 686 women using data collected from May 19, 2020 to October 3, 2020. Enrolled respondents completed assessments in which they self-reported maternal-fetal bonding, mental health symptomatology, psychological factors, and COVID-19-related worries regarding health, pregnancy, and resources.

**Results:**

Depressive symptoms in pregnant women were associated with lower quality maternal-fetal bonding, while a higher level of anxiety was positively associated with bonding; however, past history of depression or generalized anxiety diagnosis did not appear to be as relevant as active symptomatology. Maternal resilience, but not distress tolerance, appeared to be a protective factor resulting in improved bonding. Higher levels of worry regarding impact of COVID-19 on health were significantly associated with improved bonding, while worries regarding the impact of COVID-19 on the pregnancy or resources were not significantly associated with bonding. The study also found associations between different sociodemographic variables and bonding, including a strong positive association between first time motherhood and bonding and a negative association between higher education and income and bonding.

**Conclusions:**

This study was the first to report potential protective and risk factors to the maternal-fetal bonding process in women pregnant during the COVID-19 pandemic. Unique COVID-19 concerns exist; however, anxiety and COVID-19 concerns do not appear to undermine maternal-fetal bonding while active depressive symptomatology may negatively influence bonding; interventions increasing maternal resilience may be particularly valuable.

## Background

The Coronavirus Disease 2019 (COVID-19) has presented unique stressors on the pregnant population since its emergence as a global pandemic in early 2020 [1]. The direct physical effects of COVID-19 on pregnancy and on pregnant women are not well established. While the research on COVID-19 has now yielded results that are largely reassuring with regard to vertical transmission risk [2–4], recent studies point to increased morbidity and mortality of the virus in pregnant women as well as increased risk of preterm birth in infected women [5]. Beyond concerns related to the direct effects of the virus, alterations in prenatal care [6], various life stressors including the loss of employment and other resources due to the pandemic [7, 8] are major psychosocial stressors to expectant families that can exacerbate stress and mental health concerns in the family [9–12]. Although there is published work on postpartum experiences of maternal bonding during the pandemic [13–16], the maternal experience of bonding with their unborn children is limited despite COVID-19-related emotional experiences [17].

Maternal-fetal bonding, the process of forming a relationship and an emotional connection to the fetus [18, 19], is an important first step in the process of bonding between mothers and children. Choosing a name, envisioning the baby’s future, and describing the baby’s ‘personality’ based on fetal movement patterns could all be thought of as part of this transition, in addition to tangible and perceptual experiences like listening to the heartbeat on a Doppler fetal monitor and visualizing the baby on the ultrasound [20, 21]. Maternal-fetal bonding has been shown to predict attention to prenatal care [22] and postpartum bonding [23–25], an important factor considered foundational for later family and developmental outcomes [26–29]. Yet, there are few studies examining the factors associated with maternal-fetal attachment itself. Under non-pandemic conditions, a first pregnancy [30], later gestational age [24], ultrasound visualization of the fetus [31], and better partner support [32] appear to promote maternal-fetal bonding, while higher maternal education levels [32], depressive symptoms [32, 33], and substance abuse history [30] are potential risks to successful bonding; the influence of anxiety on maternal-fetal bonding has received mixed reports [19, 22, 34]. Individual characteristics such as psychological resilience or distress tolerance are thought to have a role on one’s well-being during the COVID-19 pandemic [35–37] but have not been well-studied even under non-pandemic conditions [38]. Poor maternal mental health can lead to difficulties in this bonding process and existing evidence suggests that the rates of anxiety and depression among pregnant women have increased during the pandemic beyond an already high general prevalence [39].

This study aimed to examine the associations between mental health history, current mental health symptoms, maternal psychological factors, worries related to the COVID-19 pandemic, and self-reported maternal-fetal bonding in pregnant women.

## Methods

### Study design

This was an online, survey-based cross-sectional study focused on women pregnant during the pandemic and at the time of data collection. Details of the broader study design and methodology can be found elsewhere [10, 16].

### Study setting

Recruitment and data collection took place online between May 19, 2020 and October 3, 2020; recruitment was national and utilized word of mouth, email list-serves, and social media.

### Participants

Participant eligibility criteria were female gender, aged 18 years or older, living in the United States, at least 6 weeks pregnant at the time of enrollment and data collection.

### Ethics

Prior to any data collection, those eligible and interested in participating were asked to review an overview of the study including procedures, risks, benefits, participant rights, and study team contact information. Those who remained interested in study participation were asked to provide informed written consent of the study. This was obtained by participants selecting the statement “I agree to participate in the study” at the end of the informed consent. No compensation was provided for enrollment in or completion of the study. Participation was self-selecting and opt-in, and participants were able to withdraw their participation at any time.

All procedures were approved by the Mass General Brigham Institutional Review Board.

### Data collection

Study participation constituted enrolled respondents participating in a 30-to-45-min survey administered online through REDCAP about pregnancy, experiences related to COVID-19, and psychological well-being as part of the PEACE (Perinatal Experiences and COVID-19 Effects) 2020 Study. Participants completed the survey on average 121 days after March 13, 2020, at which the pandemic was declared a national emergency in the United States. Data quality was ensured by embedding various human verification steps such as reCAPTCHA as well as number counting tasks, and attention checks, including three multiple choice questions with the correct answer being purposely against common sense that required close reading comprehension of the prompt, in addition to inspections to assess for any response irregularities among various measures. These checks were designed to ensure that answer choices throughout the study were not made at random or insincere.

### Bias

Attempts to address potential bias introduced by a self-selecting patient sample included lowering barriers to participation to encourage as widespread and representative enrollment as possible. In the United States, 85% of women own a smartphone, and over 91% of adults own either a computer or a smartphone [40, 41]. By utilizing an online rather than clinical site-based enrollment approach and by using a variety of different online forums to reach potential participants, we removed potential obstacles to participation such as disparate access to transportation and medical care. The survey was designed such that respondents could complete the study over multiple sittings to further improve ease of participation and to enable those with more time-consuming work or home responsibilities to participate.

### Study size

The study size was determined by the number of responses to the online survey during the study period. Data from all participants meeting eligibility requirements, with survey responses passing data quality standards and complete responses to all variables were used in the analysis (*N* = 686).

### Data sources

All data for the variables used in this analysis were derived from the PEACE (Perinatal Experiences and COVID-19 Effects) 2020 Study as detailed above. This dataset is proprietary but can be made available upon reasonable request to the lead author.

### Study variables

#### Predictors

##### COVID-19-related health worries

This factor was calculated as a sum score of four items from the Coronavirus Health Impact Survey (CRISIS) that were designed to evaluate an individual’s concern about COVID-19 directly affecting the health of oneself, family, and friends [42] through infection or through an influence on physical or mental health. Cronbach’s α for measure items indicated good reliability (α = 0.875).

##### COVID-19-related pregnancy worries

COVID-19-related pregnancy worries, an 8-item measure developed for this study, evaluated worries specific to pregnancy during the COVID-19 pandemic. Participants indicated their level of concern about the potential impact of COVID-19 on their pregnancy, including concerns related to vertical transmission, prenatal care, and birth partner restrictions. The measure also incorporated their self-reported stress about visiting the hospital due to the COVID-19 pandemic. Cronbach’s alpha indicated good reliability (α = 0.810).

##### COVID-19-related resource worries

COVID-19-related resource worries, a 6-item measure [43], evaluated the intensity of worries specific to accessing general resources during the COVID-19 pandemic. Participants indicated their level of concern about topics such as obtaining groceries, accessing healthcare, affording rent and basic necessities, and maintaining employment during the pandemic. Cronbach’s alpha indicated good reliability (α = 0.744).

##### Resilience

The 10-item Connor-Davidson Resilience Scale [44] was used to measure participants’ psychological resilience by assessing self-reported ability to cope with adverse experiences over the past month. Examples of items on the measure include “I am able to adapt when changes occur,” “Having to cope with stress can make me stronger,” and “I think of myself as a strong person when dealing with life’s challenges and difficulties.” Higher scores indicate higher resilience.

##### Distress tolerance

The 15-item Distress Tolerance Scale [45] was used to measure participants’ distress tolerance. Examples of items on the measure include “I’ll do anything to avoid feeling distressed or upset” and “I feel ashamed of myself when I feel distressed or upset.” Higher scores indicate greater distress tolerance.

##### Life events

The Life Events measure inquired about participant experience over the past 6 months with 13 events, using a binary ‘yes’ or ‘no’ response. Examples of items on the measure include job loss, deployment, divorce, jail time, and homelessness [46].

##### Depression symptoms

The Center for Epidemiologic Studies-Depression (CES-D)‘s 20- item self-report measure was used to evaluate the severity of depression in participants [47]. When summed, total scores range from 0 to 60 and higher scores indicate more depressive symptoms.

##### Anxiety symptoms

The Generalized Anxiety Disorder Scale (GAD-7 [48]) was used to evaluate current anxiety symptoms in participants. This 7-item scale assesses the frequency of anxiety symptoms experienced in the past two weeks using participant self-report. When summed, total scores range from 0 to 21 and higher scores indicate higher anxiety levels.

#### Outcomes

##### Maternal-fetal bonding

To assess maternal-fetal bonding, we used the Maternal Antenatal Attachment Scale (MAAS [49]). This 18-item measure inquires about thoughts and feelings about the developing baby. Scores for all measures were summed; reverse scoring was performed for some items prior to summation as required by scale. Good reliability was verified by Cronbach’s α for measure items (α = 0.753).

#### Potential confounders

##### Pandemic duration

The duration of the pandemic was defined as the period of time, in days, between when each respondent started the survey and the date when COVID-19 was declared as a pandemic (March 13, 2020).

##### Sociodemographic background

Maternal Age, Maternal Race, Maternal Education level, Maternal Income, Gestational age of the fetus, Past Psychiatric History of Major Depressive Disorder or Anxiety, and Nulliparity were also included in the analysis as covariates.

### Quantitative variables

Probable clinical depression was defined as CES-D > 16, consistent with recommendations for a cutoff score [50], with probable clinical anxiety was defined as GAD-7 > 10, in keeping with the cutoff score for moderate anxiety recommended by developers of the measures [48]. ‘High levels of worry’ for individual items within variable measures were defined as a response of ‘Worried’ or ‘Very Worried’, corresponding to the two highest options on a 5-point Likert scale. All other quantitative variables were handled as raw scores for all statistical analysis.

### Statistical methods

Data were analyzed in SPSS version 24 using descriptive and inferential statistical methods including Student’s t-test, Pearson’s correlation coefficient, and multiple regression analysis. For all analyses, only pregnancies with complete data on all variables of interest for this study were included, such that the total sample size for all analyses is constant (*N* = 686). Multiple regression analysis was conducted with maternal-fetal bonding regressed on sociodemographic characteristics (Block 1), mental health history (Block 2), current mental health symptoms (Block 3), psychosocial factors (Block 4), and COVID-19-related worries (Block 5), such that the contributions of potential confounders of maternal-fetal bonding were controlled via a blocked structure to the analysis. The variables were normally distributed and predictors indicated acceptable levels of collinearity (VIF < 5). Sensitivity analyses were not performed. Two subgroups were examined in more detail: those reporting high levels of worries about COVID-19 influencing their mental or emotional health, and those reporting high levels of worry that COVID-19 will affect their ability to bond with their baby, with CES-D and GAD-7 scores compared to the remainder of the cohort in the former subgroup, and MAAS scores in the latter subgroup, and analyzed in both cases with Student’s t-tests.

## Results

Table 1 presents the descriptive characteristics of our sample. The mean age of the sample was 33.1 years, older than the U.S. population average of 27 years [51]. In general, the sample comprised of White and college educated women. Among the respondents, 42% reported a household income of over $150,000 per year and would thus be considered to be in an ‘upper middle class’ or ‘rich’ social class [52]. Gestational age (GA) was 28.4 weeks on average, and this was the first pregnancy for 53.1% of women in this study, compared to an approximately 38% rate of first time live birth for women in the United States [51]. Approximately 17.8% of patients had a prior diagnosis of Depression while 27.0% had a prior diagnosis of Generalized Anxiety Disorder.

**Table 1:** Demographic characteristics from Wave I of the PEACE Study, data collected between May 19 and October 3, 2020

Predictors	Means (Range) or %
Maternal age (years)	33.1 (22.3–46.3)
Gestational age of fetus (weeks)	28.4 (7–41)
Maternal race	
White	92.1%
Black or African American	1.0%
Hispanic or Latino	3.6%
Asian and Pacific Islander	3.2%
Other	0.0%
Education	
Less than college	8.0%
College	31.8%
Masters	41.4%
Doctorate	18.8%
Income	
<$74,999	13.8%
$75,000-149,999	44.6%
$150,000-224,999	25.5%
>$225,000	16.0%
Prior Depression Diagnosis	
No	82.2%
Yes	17.8%
Prior Anxiety Diagnosis	
No	73.0%
Yes	27.0%
First pregnancy	
No	53.1%
Yes	46.9%
Pandemic duration (days)	121.2 (69–201)

*N* = 686

The mean scores for depression and anxiety symptoms assessed from standardized measures (CES-D, GAD-7) are shown in Table 2. We observed a mean score of 14.31 (SD = 9.04) on the CES-D and a mean score of 6.34 (SD = 4.97) on the GAD-7 (probable clinical depression and anxiety is indicated with CES-D > 16 and GAD-7 > 10, respectively). The mean score of 14.31 on the CES-D corresponds to participants reporting that they have experienced the majority of response items for 1–4 days over the past week, with each item representing a depressive symptom. A mean score of 6.34 on the GAD-7 may be conceptualized as representing a response of ‘several days’ over the past two weeks for most items relating to anxiety symptom frequency, or ‘nearly every day’ for several items. Using the aforementioned binary cutoffs, 37.2% of respondents reported symptom levels indicating probable clinical depression and 24.2% reported symptom levels indicating probable clinical anxiety. These states were highly comorbid, with 83.1% of women with probable clinical anxiety also experiencing probable clinical depression and 54.1% of women with probable clinical depression testing above the binary threshold for anxiety as well. Among our sample, 71.4% of women with probable clinical depression had no prior history of diagnosed depression, while 54.8% of women with probable clinical anxiety had no prior history of diagnosed anxiety.

**Table 2:** Maternal mental health, COVID-related concerns, and maternal experiences from Wave I of the PEACE Study, data collected between May 19 and October 3, 2020

Target Variables	Means (SD)
*Mental health symptoms*	
Depression (CES-D)	14.31 (9.04)
Generalized anxiety (GAD-7)	6.34 (4.97)
*Psychosocial factors*	
Life Events	1.35 (1.33)
Resilience (CD-RISC)	27.44 (6.13)
Distress Tolerance (DTS)	53.68 (11.99)
*COVID-19-related worries*	
Health	12.02 (3.70)
Pregnancy	20.15 (6.42)
Access to resources	11.48 (4.24)
*Maternal-fetal bonding*	
Attachment (MAAS)	71.82 (7.29)

*N* = 686

The mean number of potentially distressing life events was 1.35 (SD = 1.33), with the three most commonly experienced life events being ‘I argued with my husband or partner more than usual,’ ‘My husband, partner, or I had a cut in work hours or pay,’ and ‘A close family member was very sick and had to go to the hospital.’ The average score for Resilience was 27.44 (SD = 6.13), and for Distress Tolerance was 53.68 (SD = 11.99). The mean scores for COVID-19-related worries were 12.02 (SD = 3.70) for health worries, 20.15 (SD = 6.42) for worries regarding impact on pregnancy, and 11.48 (SD = 4.24) for worries regarding access to resources. Finally, participants had an average sum 71.82 (SD = 7.29) out of a total possible score of 90 on the MAAS; a sum of 71 or below, corresponding to less than 80% the maximum MAAS, may be interpreted to represent low antenatal bonding [53]. The responses for COVID-19-related worries were further examined in Table 3, with a response of ‘Worried’ or ‘Very Worried’ being considered to represent high levels of concern. More than two-thirds of women reported high levels of stress about going to the hospital, and over half were highly worried about their physical health being affected by COVID-19. In contrast, high levels of concern about being able to obtain testing, hospital access, and treatment for COVID-19 were relatively uncommon. Amongst pregnancy-specific worries, the most common was that the birth partner or support person would not be able to be present during labor and delivery, with 44.2% of women expressing high levels of worry. Also frequent were high levels of worry about transmitting COVID-19 to the baby during breastfeeding or caretaking, which affected 33.7% of the cohort, and worries about contracting COVID-19 during labor and delivery, which affected 29.2%. Although scientific knowledge about the risk of transmitting COVID-19 from mother to baby increased over time during the pandemic, there was no correlated decrease in these worries over time (*p* = 0.701).

**Table 3:** Maternal COVID-19-related worries

***COVID-19 Health Worries***	**‘Lower worry’**	**‘Higher worry’**
… about being infected?	84.5%	15.5%
… about friends or family being infected?	88.6%	11.2%
… about your physical health being influenced by COVID-19?	48.4%	51.6%
… about your mental/emotional health being influenced by COVID-19?	75.1%	24.9%
***COVID-19 Resource Worries***	**‘Lower worry’**	**‘Higher worry’**
...that I won’t have enough groceries during city lockdowns/social distancing protocols	93.7%	6.3%
...that I will not be able to obtain a COVID-19 test if I become sick	96.2%	3.8%
...that I will not be able to receive treatment for COVID-19 if I contract it	94.2%	5.8%
...about keeping in touch with loved ones during social distancing protocols	75.5%	24.5%
...about maintaining employment during the subsequent economic downturn	80.5%	19.5%
...about having enough money to pay for rent and buy basic necessities	89.1%	10.9%
***COVID-19 Pregnancy-Specific Worries***	**‘Lower worry’**	**‘Higher worry’**
...to hold, care for, and (breast) feed my baby because I fear I may transmit the virus to my baby	66.3%	33.7%
...I might become very sick, and I won’t have another trusted family member or friend to care for my baby if that happens	76.5%	23.5%
...I don’t have a way to get to the hospital if I/my baby becomes sick and I need to see a doctor	97.4%	2.6%
...that COVID-19 related stress will affect my ability to bond with my baby	84.7%	15.3%
...about contracting COVID-19 during labor and delivery	70.8%	29.2%
...that I am not receiving adequate prenatal care due to COVID-19	83.4%	16.6%
...that my birth partner or support person may not be able to be with me during labor and delivery	55.8%	44.2%
I feel more stressed about going to the hospital because of COVID-19.	32.9%	67.1%

*N* = 686

Overall, women were more likely to be concerned about their mental and emotional health than about contracting COVID-19 itself. High levels of worries about COVID-19 influencing their mental or emotional health were experienced by 24.9% of women. This subset of women also had significantly higher CES-D scores (16.5 vs. 13.6, *p* < 0.0001) and higher GAD-7 scores (7.7 vs. 5.9, p < 0.0001). High levels of worries on this single item response produced a positive predictive value of 48.0% for probable clinical depression (CES-D > 16) and 35.7% for probable clinical anxiety (GAD-7 > 10). Given this study’s particular interest in bonding, groups of higher and lower levels of worries were compared for the specific response item in the COVID-19 pregnancy-specific worries scale about COVID-19 affecting their ability to bond with their unborn baby. While 15.3% (105/686) of women reported high levels of worries about their bonding, there was no significant difference in MAAS scores between the higher and lower levels of worries groups (71.84 vs 71.80, *p* = 0.959).

Table 4 reports the multiple regression results. The change in R^2^ was found to be significant (*p* < .001) for all blocks besides mental health history. GA (*β* = 0.114, *p* < 0.01), first pregnancy (*β* = .169, *p* < 0.001) and mothers reporting being Hispanic/Latino (*β* = 0.082, *p* < 0.05) appeared positively associated with bonding. Higher incomes (*β* = − 0.122, p < 0.05) and educational levels (*β* = − 0.217, p < 0.01) did not appear protective. After accounting for sociodemographic characteristics as well as the pandemic duration, history of a mental health diagnosis (depression or anxiety) did not exhibit any significant association with maternal-fetal bonding. However, with prior mental health history taken into account, higher levels of endorsed depression symptoms during pregnancy were associated with lower levels of maternal-fetal bonding (*β* = − 0.297, *p* < 0.001) and higher levels of anxiety symptoms with higher levels of maternal-fetal bonding (*β* = 0.140, *p* < 0.05). When considering depressive and anxiety symptoms, higher levels of resilience were correlated with higher levels of maternal-fetal bonding (*β* = .175, p < 0.001), while distress tolerance and disruptive life events were not significantly correlated. Lastly, reported worries about COVID-19 related health were showed to contribute to the model based on the control of sociodemographic characteristics, mental health history, mental health symptoms, and psychosocial factors. Higher levels of COVID-19-related health worries were associated with higher levels of maternal-fetal bonding (*β* = 0.160, *p* < 0.01), whereas higher levels of COVID-19-related resource worries demonstrated a trend toward lower levels of maternal-fetal bonding (*β* = − 0.083, *p* < 0.1) and pregnancy-specific COVID-19 worries were not significant (*β* = 0.063, p = *n.s.*). COVID-19-related worries accounted for 18.5% of the model variance with regards to maternal-fetal bonding.

**Table 4:** Multiple regression predicting maternal-fetal bonding based on mental health history, symptoms, psychosocial factors, and COVID-19-related worries

Total score			
Blocks of variables entered in four steps	*β*	*R*^*2*^	*ΔR*^*2*^
*1. Covariates*		0.076	0.076***
Maternal age (years)	−0.051		
Gestational age of fetus (weeks)	0.114**		
Maternal race			
(ref = White)			
Black	0.048		
Hispanic	0.082*		
Asian	−0.060^†^		
Maternal education			
(ref = less than college)			
College	−0.193**		
Masters	−0.238**		
Doctorate	−0.217**		
Maternal income			
(ref = <$74,999)			
$75,000-149,999	−0.068		
$150,000-224,999	−0.057		
≥$225,000	−0.122*		
First pregnancy (ref = no)	0.169***		
Pandemic duration	0.009		
*2. Mental Health History*		0.077	0.003
Prior Depression Diagnosis (ref = no)	0.053		
Prior Generalized Anxiety Diagnosis (ref = no)	−0.037		
*3. Mental Health Symptoms*		0.14	0.064***
Depression	−0.297***		
Generalized anxiety	0.140*		
*4. Psychosocial factors*		0.164	0.028***
Life Events	0.011		
Resilience (CD-RISC)	0.175***		
Distress Tolerance (DTS)	0.031		
*5. COVID-19-Related Worries*		0.185	0.024***
Health	0.160**		
Pregnancy	0.063		
Access to resources	−0.083^†^		

*N* = 686, †*p* < 0.1, *p < .05, **p < .01, ***p < .001

## Discussion

This study aimed to examine the association between mental health history, current mental health symptoms, psychological factors, worries related to COVID-19, and the self-reported maternal-fetal bonding experiences of pregnant women during the COVID-19 pandemic. Identifying the risks and protective factors for maternal-fetal bonding during the COVID-19 pandemic can facilitate tailored counsel to patients and clinicians.

Those completing the study were predominantly White, well-educated, and financially secure women in their early thirties experiencing their first pregnancies. When reflecting upon COVID-19-related worries, participants rarely expressed worries about their ability to obtain COVID-19 testing and treatment. However, the majority of women expressed high levels of worries about the risk posed to their personal health. Entering the hospital and the labor and delivery process were of particular concern as a source of potential exposure. Although preliminary evidence doesn’t indicate vertical transmission is a significant risk [3, 54], mothers expressed no attenuation of these worries over time in the absence of population-based evidence.

A number of factors appear to be protective of maternal-fetal bonding including greater GA, first pregnancy, those who identify as Hispanic/Latino, and high resilience scores. Higher incomes and educational levels were associated with poorer bonding. These associations were largely consistent with what has been reported in the literature under non-pandemic conditions [24, 30, 32]. Depressive symptomatology during pregnancy was highly correlated to poorer bonding even after accounting for past psychiatric history. This is highly relevant and concerning given that well over a third of respondents reported scores consistent with a positive screen for probable clinical depression. Higher levels of anxiety symptoms, on the other hand, did not appear to undermine the maternal-fetal bond in the same way, consistent with what has been reported postpartum [25, 55], and on its own appeared to be a protective factor for bonding. Similarly, COVID-19-related worries about pregnancy and obtaining resources were not negatively associated with maternal-fetal bonding and in the case of COVID-19-related health worries appeared to be protective. Even self-reported worries specifically about maternal-fetal bonding did not appear to predict poorer bonding. High levels of anxiety could in fact reflect maternal attachment and care toward their pregnancy during an uncertain time; alternatively, anxiety may predispose pregnant women to feeling more protective toward their unborn child [19].

Of psychological factors assessed by this study, resilience appeared to be a meaningful protective factor for maternal-fetal bonding. Past research has demonstrated resilience as a protective factor against prenatal anxiety and depression both in non-pandemic [56] and pandemic conditions [57], but our results suggest that resilience is associated with bonding even after accounting for depression and anxiety levels. It may be the case that women with higher resilience are more likely to take a long-term perspective of the future amenable to picturing their developing fetus as a person, or believe in their ability to develop an emotional connection. Higher levels of resilience were protective even in those with higher levels of depression. In contrast, distress tolerance was not significant, suggesting that the ability to adapt and overcome long-term challenges may be more relevant than the ability to cope with acute frustrations. Therefore, resilience-focused interventions using cognitive-behavioral therapy (CBT), mindfulness training, or a combination of both may be of particular benefit to patients during the pandemic [58–60].

Notably, past psychiatric history was not associated with bonding. Considering most women reporting probable clinical depression or anxiety had no prior diagnoses of depression or anxiety, this suggests clinically relevant distress may be newly emerging in many pregnant women and all patients should be screened carefully. Those presenting to clinics with anxiety symptoms should also be screened for depression given the high degree of comorbidity and disparate potential impacts on maternal-fetal bonding and because prenatal anxiety is associated with later postpartum depression [61].

This study has some limitations presented by the voluntary and self-selecting nature of participant enrollment, which resulted in demographics that are highly weighted toward highly educated, higher income, White women. Minimal concern regarding resource acquisition may reflect the relative financial stability of most completing the survey. These factors all may limit the generalizability of the study findings, and in the future additional focus on racial/ethnic minority and disadvantaged groups will be needed. As well, it is possible that women with COVID-19-related worries were more interested in engaging with the study, which could result in an overinflation of estimates of worry/anxiety symptoms; similarly, those with very high levels of depressive symptoms impairing concentration and attention may have been screened out by quality control attention checks and thus be underrepresented. However, the design of the study may also allow it to be particularly well-poised to capture the group of women who are most likely to be presenting questions and concerns to their obstetricians at this time.

## Conclusions

Based on these findings, we conclude that COVID-19-related worries, in addition to depressive and anxiety symptoms, may influence maternal-fetal bonding during the pandemic. This work has several clinical implications. OB/GYN providers should plan to screen all pregnant women for depression, early and often, regardless of mental health history and to speak to their patients regarding their mood [62]. Clinicians should be aware that patients with higher levels of current depressive symptoms during the COVID-19 pandemic are at risk for poorer maternal-fetal bonding, which may have implications for the subsequent maternal-infant relationship and infant neurodevelopmental outcomes. At present, the anxiety symptoms reported as well as worries specific to COVID-19 by pregnant women do not appear to be a risk for maternal-fetal bonding. Resilience may improve maternal-fetal bonding even among those with higher levels of depressive symptoms and thus provides a potential target for interventions. Finally, women who are concerned about their ability to bond with their unborn baby or that their anxiety symptoms may affect bonding may benefit from reassurance based on this study data.

## Data Availability

The dataset used and/or analyzed during the current study are available from the corresponding author on reasonable request.

## Abbreviations

COVID-19: Coronavirus Disease 2019
CRISIS: Coronavirus Health Impact Survey
CD-RISC 10: Connor-Davidson Resilience Scale 10 Items
DTS: Distress Tolerance Scale
CES-D: Center for Epidemiologic Studies-Depression
GAD-7: Generalized Anxiety Disorder Scale
MAAS: Maternal Antenatal Attachment Scale
GA: Gestational Age
CBT: Cognitive-Behavioral Therapy
